# Personal

**Published:** 1903-01

**Authors:** 


### PERSONAL.

Dr. Richard H. Satterlee, of Buffalo, has removed from 187 Delaware Avenue to The Touraine, corner of Delaware Avenue and Johnson Park. Practice limited to diseases of the eye. Hours: 9-1, 3-4.

---

Lt.Colonel G. Sterling Ryerson, M. D., of Toronto, has been promoted to the grade of colonel in recognition of his services as Red Cross Commissioner in South Africa.

---

Dr. G. Frank Lydston, of Chicago, has recently returned from an extended tour of Australia, New Zealand, Samoa and the Hawaiian Islands.

---

Dr. Frank L. Carpenter, U. of B.'91, of Vacaville, Cal., recently visited Buffalo. He was accompanied by Mrs. Carpenter and they were the guests of Dr. Robert Hebenstreit,'of 430 Broadway.

---

Dr. Charles H. Andrews, U. of B., '88, who entered the military service as acting assistant surgeon during the war with

Spain, and was subsequently transferred to the Philippines, has returned to Buffalo. His present address is 1015 Elmwood Avenue.

---

Dr. Ira C. Brown, surgeon U. S. Volunteers, who has been on duty in the Philippines for the past three years, has returned to Buffalo, where he expects to remain during the winter. His address is 788 Seventh Street.

---

Dr. H. H. Glosser, formerly of Paoli, Pa., has located in Buffalo. His office and residence are at 1007 Elmwood Avenue. Dr. Glosser will make examinations of blood, urine and sputum. Telephone, North, 1112.

---

Dr. Adolf Lorenz, professor of orthopedic surgery in the University of Vienna, has received the honorary degree of doctor of laws from the Northwestern University. The ceremony was witnessed by the trustees, faculty and several hundred invited guests.

